# Curcumin attenuates ochratoxin A and hypoxia co-induced liver injury in grass carp (*Ctenopharyngodon idella*) by dual targeting endoplasmic reticulum stress and apoptosis via reducing ROS content

**DOI:** 10.1186/s40104-024-01089-2

**Published:** 2024-10-04

**Authors:** Liangqin Wu, Piao Zhao, Pei Wu, Weidan Jiang, Yang Liu, Hongmei Ren, Xiaowan Jin, Xiaoqiu Zhou, Lin Feng

**Affiliations:** 1 Animal Nutrition Institute, Sichuan Agricultural University, Chengdu, 611130 China; 2 Fish Nutrition and Safety Production University Key Laboratory of Sichuan Province, Sichuan Agricultural University, Chengdu, 611130 China; 3 Key Laboratory of Animal Disease-Resistance Nutrition, Ministry of Education, Ministry of Agriculture and Rural Affairs, Key Laboratory of Sichuan Province, Chengdu, Sichuan 611130 China

**Keywords:** Apoptosis, Curcumin, Endoplasmic reticulum stress, Grass carp, Hypoxia, Ochratoxin A, Oxidative damage

## Abstract

**Background:**

Ochratoxin A (OTA) is a toxin widely found in aquafeed ingredients, and hypoxia is a common problem in fish farming. In practice, aquatic animals tend to be more sensitive to hypoxia while feeds are contaminated with OTA, but no studies exist in this area. This research investigated the multiple biotoxicities of OTA and hypoxia combined on the liver of grass carp and explored the mitigating effect of curcumin (CUR).

**Methods:**

A total of 720 healthy juvenile grass carp (11.06 ± 0.05 g) were selected and assigned randomly to 4 experimental groups: control group (without OTA and CUR), 1.2 mg/kg OTA group, 400 mg/kg CUR group, and 1.2 mg/kg OTA + 400 mg/kg CUR group with three replicates each for 60 d. Subsequently, 32 fish were selected, divided into normoxia (18 fish) and hypoxia (18 fish) groups, and subjected to hypoxia stress for 96 h.

**Results:**

CUR can attenuate histopathological damage caused by coming to OTA and hypoxia by reducing vacuolation and nuclear excursion. The alleviation of this damage was associated with the attenuation of apoptosis in the mitochondrial pathway by decreasing the expression of the pro-apoptotic proteins Caspase 3, 8, 9, Bax, and Apaf1 while increasing the expression of the anti-apoptotic protein Bcl-2, and attenuation of endoplasmic reticulum stress (ERS) by reducing Grp78 expression and *chop* levels. This may be attributed to the fact that the addition of CUR increased the levels of catalase (CAT) and glutathione reductase (GSH), increased antioxidant capacity, and ensured the proper functioning of respiratory chain complexes I and II, which in turn reduced the high production of reactive oxygen species (ROS), thus alleviating apoptosis and ERS.

**Conclusions:**

In conclusion, our data demonstrate the effectiveness of CUR in attenuating liver injury caused by the combination of OTA and hypoxia. This study confirms the feasibility and efficacy of adding natural products to mitigate toxic damage to aquatic animals.

**Supplementary Information:**

The online version contains supplementary material available at 10.1186/s40104-024-01089-2.

## Introduction

Ochratoxin A (OTA) is one of the most toxic of the secondary metabolites produced by *Aspergillus* and *Penicillium* species [1] and is widely present in a variety of food and aquafeed ingredients, which poses a significant health risk to fish. Meanwhile, with the continuous expansion of aquaculture scale and increasing aquaculture density, hypoxia has become a common stressor faced by aquatic animals. However, studies on the combined toxic effects of OTA and hypoxia on aquatic animals have yet to be reported; further research is needed.

The liver is the most critical detoxification organ and the most important metabolic organ in fish. The liver bears the brunt of much damage when hit by both OTA and hypoxia, so liver health plays a significant role in fighting off toxicity and damage. While there are no specific studies on the effects of the combination on liver injury, it has been shown that dietary OTA reduced the growth performance of juvenile tambaqui (*Colossoma macropomum*) [2] and caused lesions in the hepatopancreatic tissue of the channel catfish (*Ictalurus punctatus*) [3]. Hypoxia can also hinder fish growth and lead to oxidative damage in the liver [4].

Given that the dangers of OTA and hypoxia have become imminent problems, the addition of natural products, usually through the addition of feed, is a common and effective method. For example, quercetin reduced OTA-induced liver injury in mice [5], resveratrol attenuated intermittent hypoxia-induced lung injury in rats by activating the Nrf2/ARE pathway [6]. As we all know, curcumin (CUR) is also a polyphenolic compound extracted from turmeric [7], which has powerful antioxidant properties and low toxicity [8]. Curcumin is not only one of the first nine natural colorants approved by the Codex Alimentarius Commission of the Food and Agriculture Organization of the United Nations for use in the Chinese food industry, but also included in the Chinese Catalogue of Feed Additives in 2014. In grass carp, CUR could enhance the immune function, physical, chemical and immune barriers in the gills [9], and attenuate OTA toxicity to muscle in vivo and in vitro [10]. Furthermore, in Nile tilapia (*Oreochromis niloticus*), CUR protected against liver damage caused by hexavalent chromium exposure [11]. Thus, CUR may be able to mitigate the damage caused by the combination of OTA and hypoxia in grass carp, but further research is needed. This is mainly due to the mitigation of oxidative damage in fish through antioxidant action, which in turn alleviates apoptosis and endoplasmic reticulum stress (ERS).

Excess reactive oxygen species (ROS) are one of the major culprits of oxidative damage. When mitochondrial function is impaired, large amounts of ROS are produced [12]. The functional state of mitochondria is closely related to respiratory chain complex activity and ATP production. Although there are no specific studies on CUR attenuating mitochondrial dysfunction caused by the combination of OTA and hypoxia, some studies have shown that CUR alleviated OTA-induced increase in mitochondrial transcription factor A, B1 and B2 gene expression [13]. Meanwhile, CUR also prevented oxidative stress-induced mitochondrial fragmentation in astrocytes [14]. As a result, CUR may be able to alleviate the mitochondrial dysfunction in grass carp caused by these two together, thus reducing ROS production, but further studies are needed.

Oxidative damage can lead to apoptosis [15], which is mainly mediated by cytochrome c (Cytc), apoptosis-associated factor 1 (Apaf1), Caspase family, and Bcl-2 family-related proteins. Although there are no reports on whether CUR can alleviate apoptosis caused by the combination of OTA and hypoxia, related studies have shown that CUR can alleviate lithium-induced apoptosis in the thyroid by reducing the protein expression of Caspase 3 [16]. Resveratrol, also a polyphenol, can alleviated doxorubicin-induced cardiomyocyte apoptosis by increasing P53 expression, modulating Bcl-2/Bax expression, and enhancing Caspase 3 activity [17]. It indicates that CUR may alleviate the apoptosis caused by OTA and hypoxia, which needs further investigation.

Oxidative damage can also lead to endoplasmic reticulum stress [18], which is mainly mediated by activating transcription factor 6 (Atf6), protein kinase R-like ER kinase (Perk), inositol-requiring enzyme 1α (Ire1α), and C/EBP homologous protein (Chop), etc. These proteins are activated by binding-immunoglobulin protein (Bip) and glucose-regulated protein 78 (Grp78) due to the accumulation of misfolded proteins. Although it has not been reported whether CUR can alleviate the ERS caused by both, related studies have shown that chronic CUR treatment can abolish hypoxia-induced increase in the protein expression of Grp78 and Chop in rats [19] and the plant extract oleanolic acid also significantly alleviate OTA-induced ERS in renal HK-2 cells by down-regulating the protein expression of Grp78 and Chop [20]. It indicates that CUR may alleviate the ERS caused by OTA and hypoxia, which needs further investigation.

The above studies suggest that CUR may reduce oxidative damage by attenuating mitochondrial dysfunction and thereby reducing ROS production to alleviate apoptosis and ERS further. Therefore, the present study aimed to investigate whether hypoxia exacerbates OTA-induced liver injury and whether CUR with antioxidant properties mitigates the hazards by examining the liver histopathological changes, oxidative damage, mitochondrial dysfunction, apoptotic and ERS parameters of the grass carp. Thus, we can systematically understand the biohazards of OTA and hypoxia on aquatic animals and obtain effective defenses.

## Materials and methods

### Reagents source

The serum stress indicators alanine aminotransferase (ALT), aspartate aminotransferase (AST), lactate dehydrogenase (LDH), glucose (GLU), antioxidant components superoxide dismutase (SOD), catalase (CAT), glutathione (GSH) determination kit and ATP content kit were purchased from the Nanjing Jiancheng Bioengineering Institute (Nanjing, China). The serum cortisol (Cor) levels were measured by using the enzyme-linked immunoassay for fish species. The ROS kit was purchased from Beyotime (Shanghai, China). RNAiso Plus kit was purchased from TaKaRa Bio (Japan). Primers were synthesized by Chengdu Youkang Jianxing Bio-technology Co., Ltd. (China), the specific information of the primary antibody used in Western blot is shown in Table S1. β-Actin (Abclonal, China, 1:3,000) was chosen as the internal reference protein, and the secondary antibody was also purchased from Abclonal and diluted with 1:8,000. TUNEL Apoptosis Detection kit was purchased from Boster (Wuhan, China). Immunol Fluorence Staining kit with Alexa Fluor 488 and 555-Labeled Goat Anti-Rabbit IgG were purchased from Beyotime, and the specific information of primary antibodies used for immunofluorescence is shown in Table S2.

### Diet design

The animal research protocol was approved by the Animal Care Advisory Committee of Sichuan Agricultural University (Chengdu, Sichuan, China).

The basal diet is shown in Table S3. Juvenile grass carp were purchased from Deyang City, Sichuan Province. After 2-week acclimatization period, 720 healthy fish with an initial weight of 11.07 ± 0.07 g were randomly divided into 4 groups of 3 replicates each and put into 12 net cages of 60 fish each. OTA and CUR were added to the diets to form four diets of 0 (no addition), 1.2 mg/kg OTA, 400 mg/kg CUR, and 1.2 mg/kg OTA + 400 mg/kg CUR. The amount of OTA and CUR added was obtained from our previous studies [21, 22]. And then conducted a 60-d feeding experiment, during which the water temperature was maintained at 28 ± 2 °C, pH was 7.5–8.0, and the dissolved oxygen was above 6.0 mg/L. The final average weight of the four groups were 152.59 ± 5.12 g, 93.94 ± 2.23 g, 200.97 ± 2.79 g, and 131.22 ± 3.69 g.

**Fig. 1:**
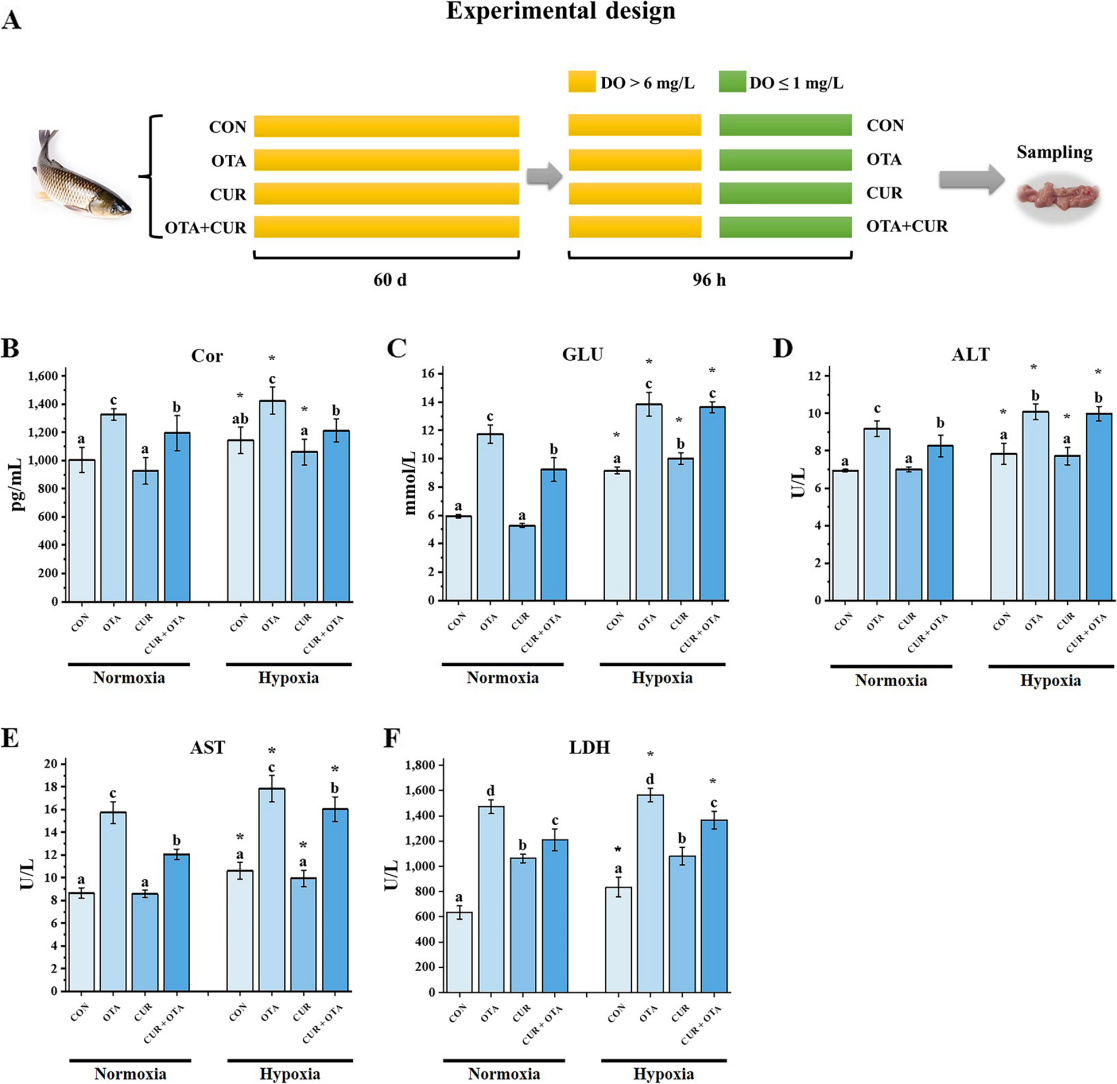
Experimental design diagram (**A**) and liver serum biochemical indices (**B–F**). Data represent means of each group; error bars indicate SD. *n* = 6. Different letters denote significant differences between normoxia or hypoxia groups (*P* < 0.05), respectively, and * denotes significant differences between normoxia and hypoxia groups at the same level (*P* < 0.05). Cor: cortisol; GLU: glucose; ALT: alanine aminotransferase; AST: aspartate aminotransferase; LDH: lactate dehydrogenase

At the end of the feeding experiment, 32 fish were selected from each net cage and divided into normoxia (18 fish) and hypoxia (18 fish) groups for a 96-h hypoxia stress experiment. The normoxia group was kept at the normal oxygen level (above 6.0 mg/L) and the hypoxia group was controlled at 1 mg/L with a timer. The specific experimental design is shown in Fig. 1A. Furthermore, the level and duration of hypoxia according to Wang’s study [23].

### Sample collection

At the end of the 96-h hypoxia stress experiment, blood was taken from the tail vein and centrifuged at 3,000 × *g* for 10 min at 4 °C to obtain serum samples. The fish were then anaesthetized in a benzocaine bath (50 mg/L) and then executed as described by Dong et al. [24]. Meanwhile, the visceral mass was immediately dissected on an ice-cold plate to strip the liver tissue. Liver tissues were preserved in liquid nitrogen, 4% paraformaldehyde, and 2.5% glutaraldehyde, respectively.

### Histological and ultrastructural observation

#### Histological observation

Histological observations refer to He’s study [25]. A small portion of the liver sample was removed from the fish and immediately rinsed with saline. It was then stored in 4% paraformaldehyde for fixation. The tissue samples were progressively dehydrated with ethanol (75%, 85%, 95%, 100%) and then paraffin-embedded. The wax blocks were then cut into thin slices using the Leica slicer, and after Hematoxylin and Eosin (H&E) staining, the tissue sections were observed at 200× using an OLYMPUS BX43 light microscope.

#### Ultrastructural observation

Ultrastructural observations refer to Liu’s study [26]. A small piece of fresh liver tissue with minor mechanical damage (e.g., contusion and crush) was selected and fixed in 2.5% glutaraldehyde. After further fixation in 1% osmium acid protected from light, it was dehydrated with ethanol and acetone in steps (ethanol: 30%-50%-70%-80%-95%-100%-100%; acetone: 100%-100%). They were then osmotically embedded with an epoxy resin embedding agent. Ultrathin sections were cut with a diamond knife and then stained with uranium acetate and lead citrate for 8 min. Finally, the sections were observed under a transmission electron microscope (HT7800, Servicebio, China), and images were collected for analysis.

### Analysis of serum and liver tissue biochemical parameters

Serum biochemical indices of Cor, GLU, ALT, AST, and LDH were measured by kit using commercial methods. Liver samples were homogenized in saline at ice-cold temperature; then the tissue homogenate was centrifuged at 6,000 × *g* for 20 min at 4 °C. And the supernatant was separated to assay the relevant enzyme activities. Antioxidant-related enzyme indicators, such as SOD and CAT, non-enzymatic such as GSH, oxidative damage indicators ROS and ATP content were measured by kit using commercial methods.

### TUNEL analyses

The TUNEL experiment was performed as follows: Paraffin sections were deparaffinized and tissues were repaired with proteinase K. The liver cells were then membrane-broken and buffer incubated at room temperature before labeling. Then, the nuclei were restained with DAPI to seal the sections with anti-fluorescence quenching sealer. Sections were observed under a DMI4000B inverted fluorescence microscope, keeping the settings of the inverted fluorescence microscope the same for all subsequent images. Three images were acquired at 200× objective for each treatment. Images were thresholded to exclude background fluorescence and were gated to contain only intensity measurements of positively stained cells. TUNEL relative fluorescence intensity was calculated using the formula: Integrated Density of TUNEL positively stained cells/Area of DAPI staining × 100. The resulting ratio was normalized to 1 in the control normoxia group.

### Real-time quantitative polymerase chain reaction (RT-qPCR) analysis

RT-qPCR analysis was performed as follows: Total RNA was isolated from the liver using an RNAiso Plus kit (Takara, Dalian, China). At the same time, the purity and content were detected by agarose gel electrophoresis (1.5%) and (A_260/280_) spectrophotometry respectively. The cDNA synthesis kit was used to reverse-transcribe RNA to form cDNA [27]. All primer sequences used in this study are shown in Table S4. The results are represented as matrix bubble plots.

### Western blot analysis

Western blot analysis was performed as follows: The liver tissues were ground using a tissue grinder after the addition of RIPA Lysis Buffer (Beyotime, China). Using a BCA assay kit (Beyotime, China), we measured the protein concentration of the supernatant. Liver samples were separated by SDS-PAGE (10%) and transferred onto a PVDF membrane using a wet Trans-Blot system (Bio-Rad, USA). After sealing, the membrane was incubated with primary and secondary antibodies. Protein signals were visualised using the ECL kit (Beyotime, China) in a high-sensitivity chemiluminescent imaging system (Bio-Rad, USA). The grey scale values of each band were obtained using Image J, 1.54*f* (NIH Image J system, Bethesda, MD, USA). The relative expression levels of each protein were normalized using the internal reference protein (β-actin) and the resulting ratio was normalized to 1 in the control normoxia group.

### Immunofluorescence staining

The immunofluorescence staining experiments were performed as follows: Paraffin sections were deparaffinized with dewaxing solution, and then the sections were placed in 3% hydrogen peroxide to remove catalase. Thermal antigen repair was then performed in a microwave oven. Primary antibodies were incubated overnight at 4 °C and washed thrice with PBS. Afterward, secondary antibodies were incubated and washed in PBS, followed by DAPI staining, PBS washing, and dropwise sealing of the sections with an anti-fluorescence quencher. The stained sections were observed using a DMI4000B inverted fluorescence microscope. The settings of the inverted fluorescence microscope were kept the same for all subsequent images. Six images per treatment were acquired at 200× objective. Images were thresholded to exclude background fluorescence and gated to contain only intensity measurements of positively stained cells. Relative fluorescence intensity was calculated using the formula: Integrated Density of positively stained cells/Area of DAPI staining × 100. The resulting ratio was normalized to 1 in the control normoxia group.

### Statistical analysis and plotting

SPSS 19.0 was used for data analysis. Data from normoxia and hypoxia groups were analyzed separately by one-way ANOVA and compared using Duncan’s multiple comparison test. The student’s *t*-test was used to compare the normoxia and hypoxia groups. Differences were considered statistically significant at *P* < 0.05. Results were expressed as mean ± standard deviation (SD). After data analysis was completed, all figures were plotted using Origin 2021.

## Results

### Effect of curcumin on liver serum biochemical indices induced by the combination of ochratoxin A and hypoxia stress

The serum biochemical parameters of grass carp are shown in Fig. 1B. Compared with the normoxia group, the GLU level, and ALT and AST activity, were significantly higher in the hypoxia group (*P* < 0.05). The activity of LDH in the control, OTA, and CUR + OTA groups was significantly higher in the hypoxia group than those in the normoxia group (*P* < 0.05), and the level of Cor in the control, OTA, and CUR groups was significantly higher in the hypoxia group than those in the normoxia group (*P* < 0.05). Besides, hypoxia significantly exacerbated the upregulation of Cor, GLU, ALT, AST, and LDH levels induced by OTA (*P* < 0.05). Immediately following this, we performed a one-way ANOVA and found that adding CUR significantly reduced the levels of Cor, AST, and LDH both under normoxia and hypoxia (*P* < 0.05).

### Effect of curcumin on liver morphology induced by the combination of ochratoxin A and hypoxia stress

**Fig. 2:**
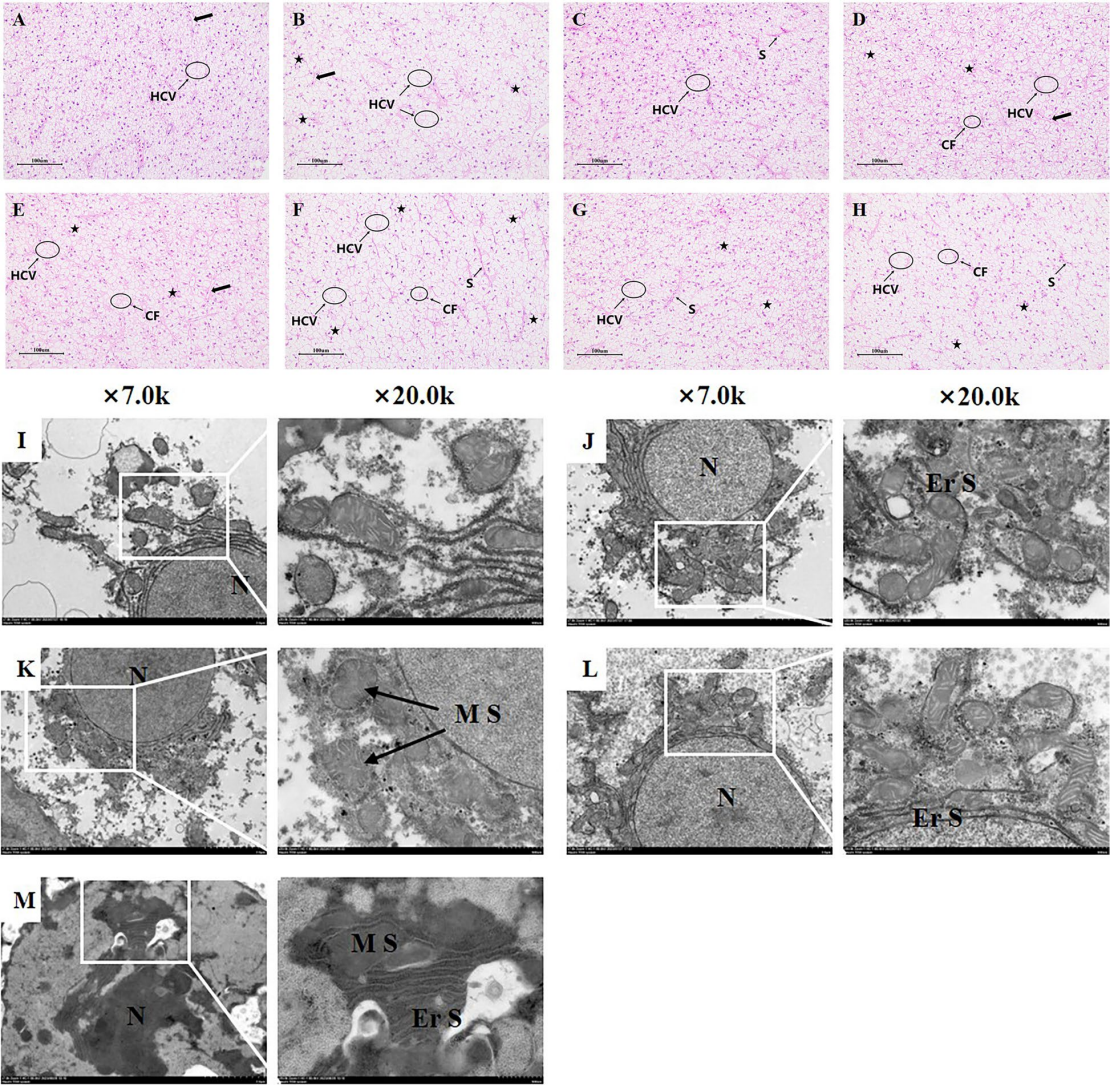
The histology in the liver of grass carp. **A**–**H** H&E, scale bar = 100 μm (× 200). S: haematological sinusoids; thick arrows: haematological sinusoids were congested and dilated; ★: cell nuclei were shifted; HCV: vacuolation chemotaxis; CF: cell fusion. **I**–**M** TEM, scale bar = 2 μm and 500 nm. N: nucleus; Er S: endoplasmic reticulum swelling; M S: mitochondrial swelling. **A** and **I** Control normoxia group; **B** OTA normoxia group; **C** CUR normoxia group; **D** OTA + CUR normoxia group; **E** and **J** Control hypoxia group; **F** and **K** OTA hypoxia group; **G** and **L** CUR hypoxia group; **H** and **M** OTA + CUR hypoxia group

We observed the liver by HE staining (Fig. 2A) and found that the liver cells in control and CUR groups were well-aligned and well-defined, but a small number of liver cells were seen to be vacuolated. In the OTA group, multiple vacuolations could be observed, with a small number and density of cells, shifted nuclei, and the occurrence of congestion and dilatation of blood sinuses. Meanwhile, after hypoxia treatment, the OTA group had the most severe damage with vacuolation of liver cells in many places, as well as nuclei shifting and cell fusion. However, the addition of CUR provided relief from liver injury caused by OTA and hypoxia.

The results of transmission electron microscopy (Fig. 2B) showed that the control normoxia group was relatively normal; the mitochondria and endoplasmic reticulum of the control hypoxia group and the CUR hypoxia group were slightly swollen with transparent cristae. However, the mitochondria of the OTA hypoxia group were severely swollen, with structural disruption, reduced number, fuzzy or even disappeared cristae, and fuzzy morphology of the endoplasmic reticulum. Yet the morphology of mitochondria of the CUR + OTA hypoxia group was somewhat alleviated compared with that of the OTA group, with the cristae still fuzzy and the endoplasmic reticulum clear but slightly swollen.

### Effect of curcumin on liver oxidative damage induced by the combination of ochratoxin A and hypoxia stress

**Fig. 3:**
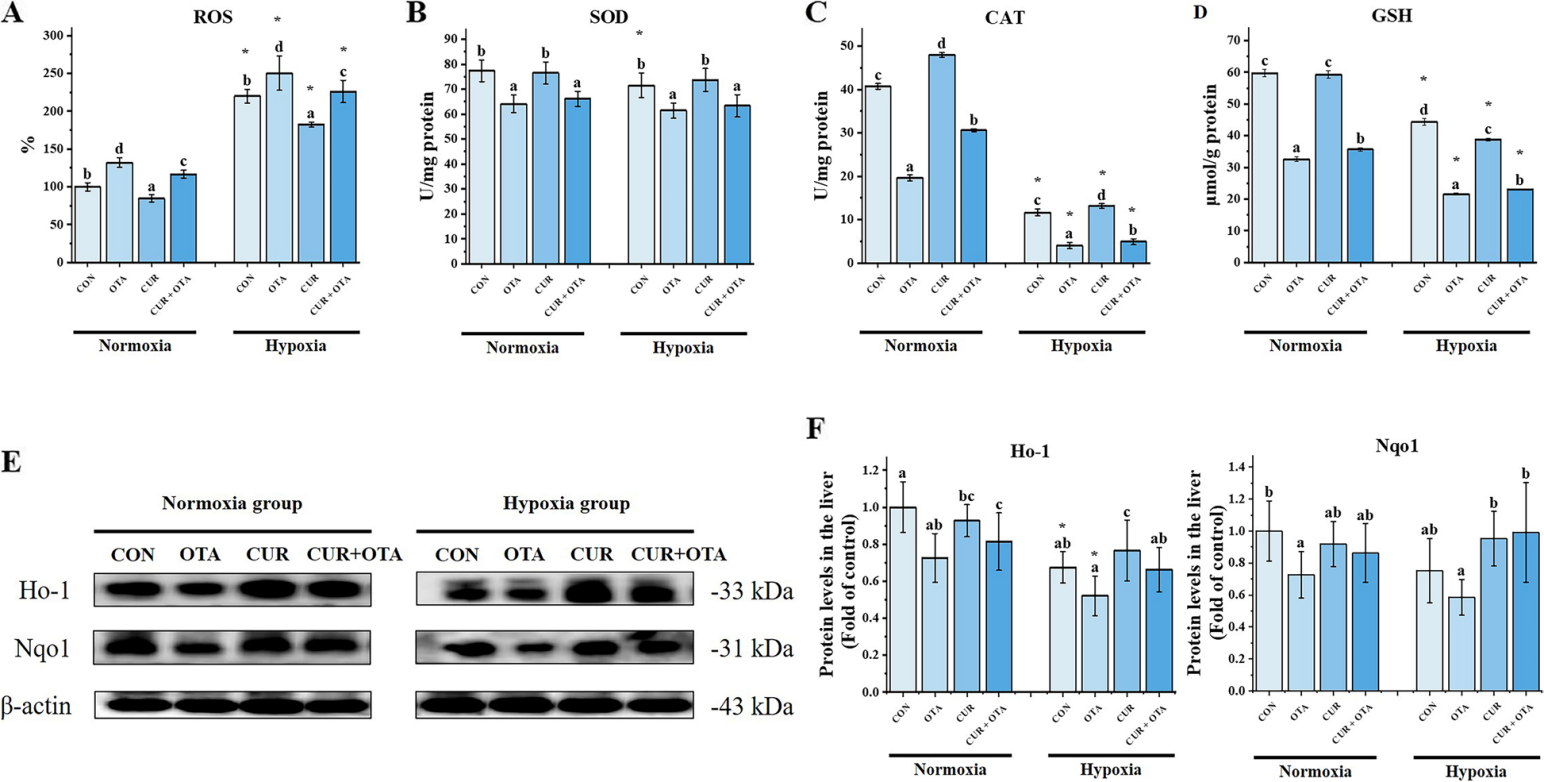
Changes in liver oxidative damage levels and antioxidant-related enzyme activities. Data represent means of each group; error bars indicate SD. *n* = 6. Different letters denote significant differences between normoxia or hypoxia groups (*P* < 0.05), respectively, and * denotes significant differences between normoxia and hypoxia groups at the same level (*P* < 0.05). ROS: reactive oxygen species; SOD: superoxide dismutase; CAT: catalase; GSH: glutathione reductase; Ho-1: heme oxygenase-1; Nqo1: NAD(P)H:quinone oxidoreductase 1

The parameters of oxidative damage in the liver of grass carp are shown in Fig. 3. We found that the levels of ROS in the hypoxia group were all significantly higher than in the normoxia group (*P* < 0.05). The levels of CAT and GSH in the hypoxia group were all significantly lower than in the normoxia group (*P* < 0.05), while SOD was not considerably different between normoxia and hypoxia group (*P* > 0.05). Hypoxia exacerbated the OTA-induced increase in ROS levels and the decrease in CAT activities, GSH content, and heme oxygenase 1 (HO-1) protein expression (*P* < 0.05). Then, one-way ANOVA revealed that the addition of CUR significantly down-regulated the ROS levels (*P* < 0.05) and up-regulated the CAT activities and GSH content (*P* < 0.05) both under normoxia and hypoxia.

### Effect of curcumin on liver mitochondrial function induced by the combination of ochratoxin A and hypoxia stress

**Fig. 4:**
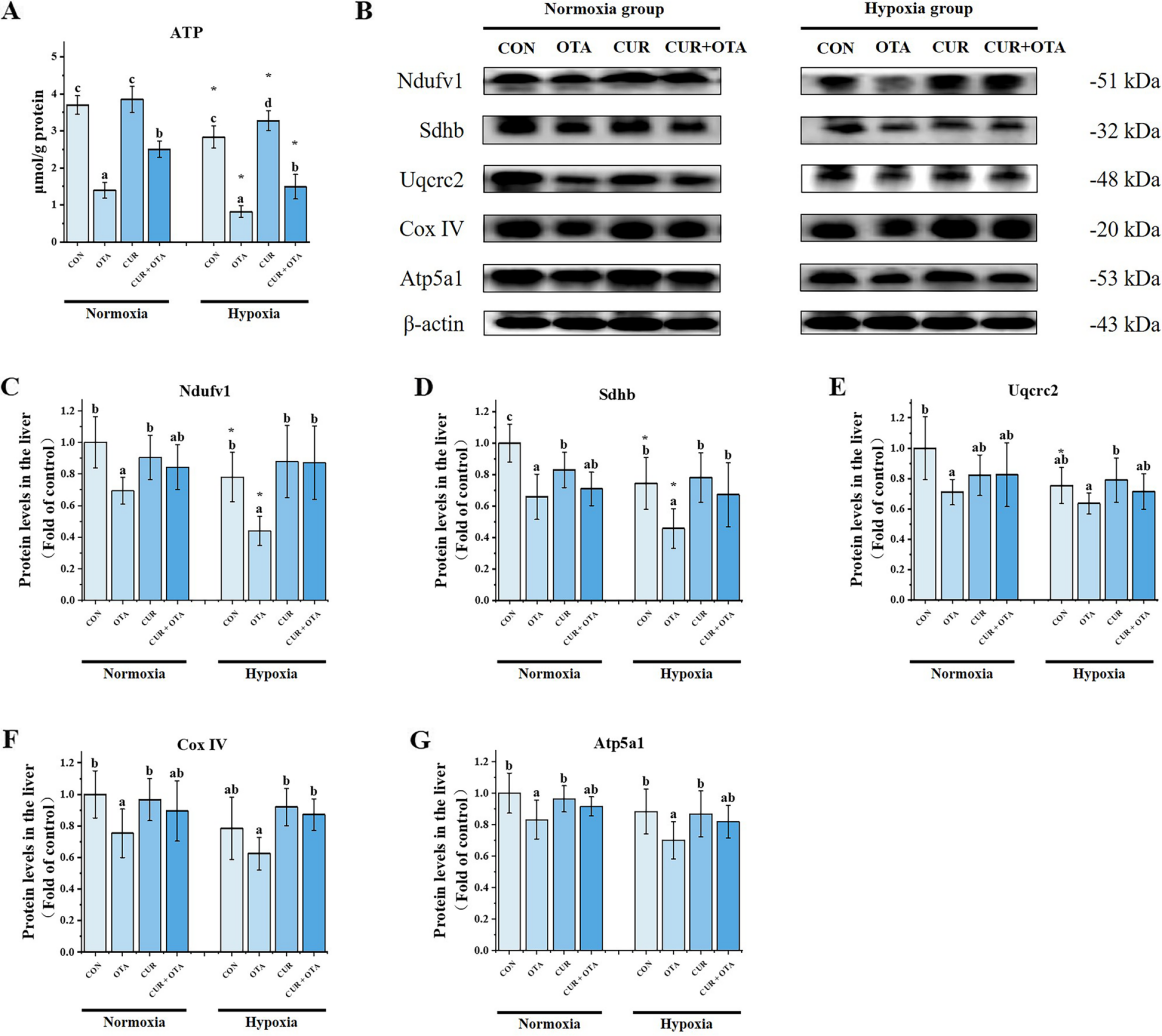
Changes in liver mitochondrial ATP levels (**A**) and mitochondrial respiratory chain complex-related protein levels (**B**–**G**) in grass carp. The control normoxia group was used as a reference. Data represent means of each group; error bars indicate SD. *n* = 6. Different letters denote significant differences between normoxia or hypoxia groups (*P* < 0.05), respectively, and * denotes significant differences between normoxia and hypoxia groups at the same level (*P* < 0.05)

Liver ultrastructure revealed that hypoxia exacerbated OTA-induced mitochondrial swelling. To further test the effect of CUR on liver mitochondrial function under the co-induction of OTA and hypoxia, we examined liver ATP levels and mitochondrial respiratory chain complex-related protein expression. As shown in Fig. 4, we found that the ATP levels of the hypoxia group were all significantly lower than the normoxia group (*P* < 0.05). Besides, hypoxia significantly exacerbated OTA-induced reduction of ATP levels and mitochondrial respiratory chain complexes I and II protein expression (*P* < 0.05). Meanwhile, One-way ANOVA revealed that the addition of CUR could up-regulate ATP levels both under normoxia and hypoxia (*P* < 0.05).

### Effect of curcumin on liver apoptosis induced by the combination of ochratoxin A and hypoxia stress

**Fig. 5:**
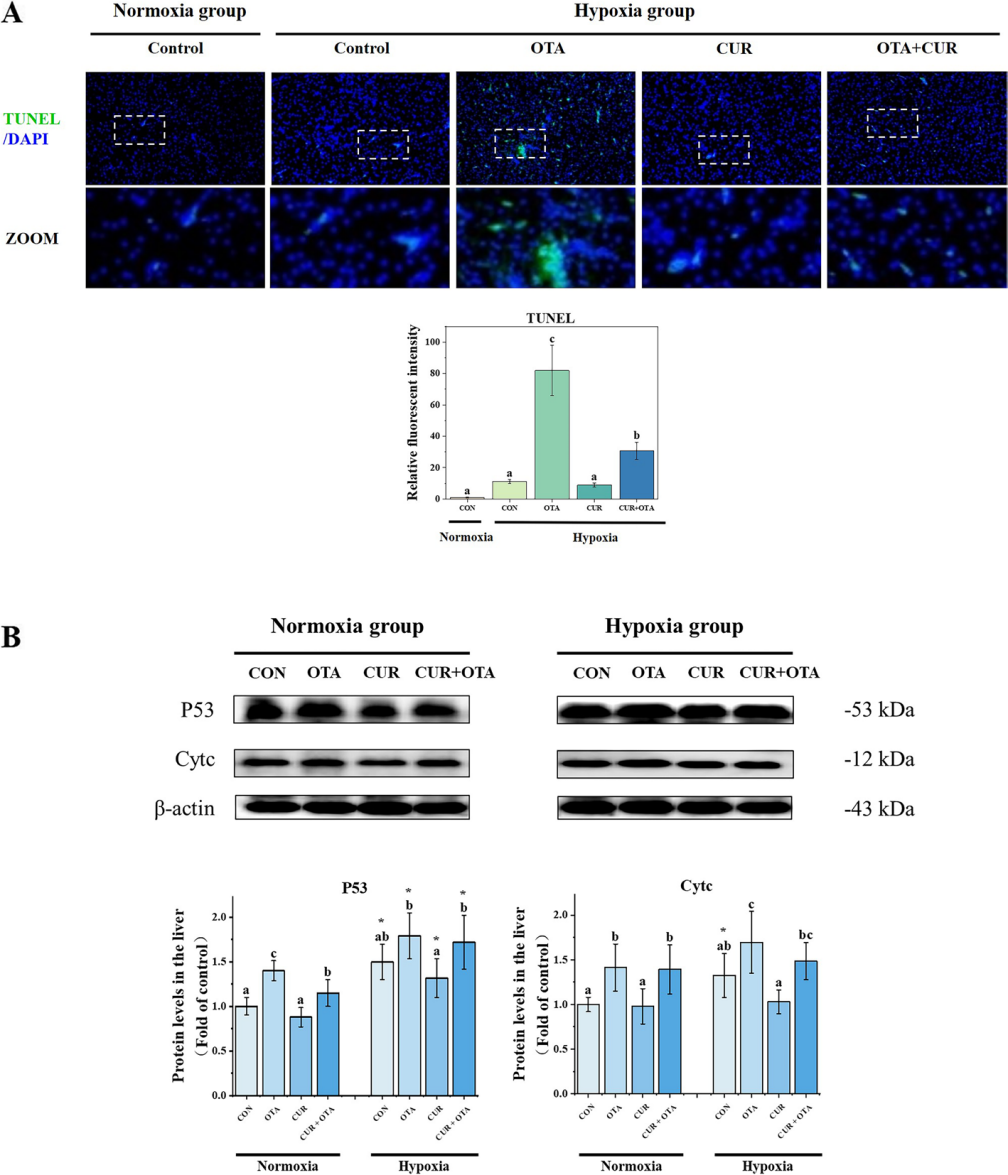
Tunel analysis (**A**) and the changes in the levels of apoptosis-related proteins (**B**) in grass carp liver. The control normoxia group was used as a reference. Data represent means of each group; error bars indicate SD. *n* = 6. Different letters denote significant differences between normoxia or hypoxia groups (*P* < 0.05), respectively, and * denotes significant differences between normoxia and hypoxia groups at the same level (*P* < 0.05)

**Fig. 6:**
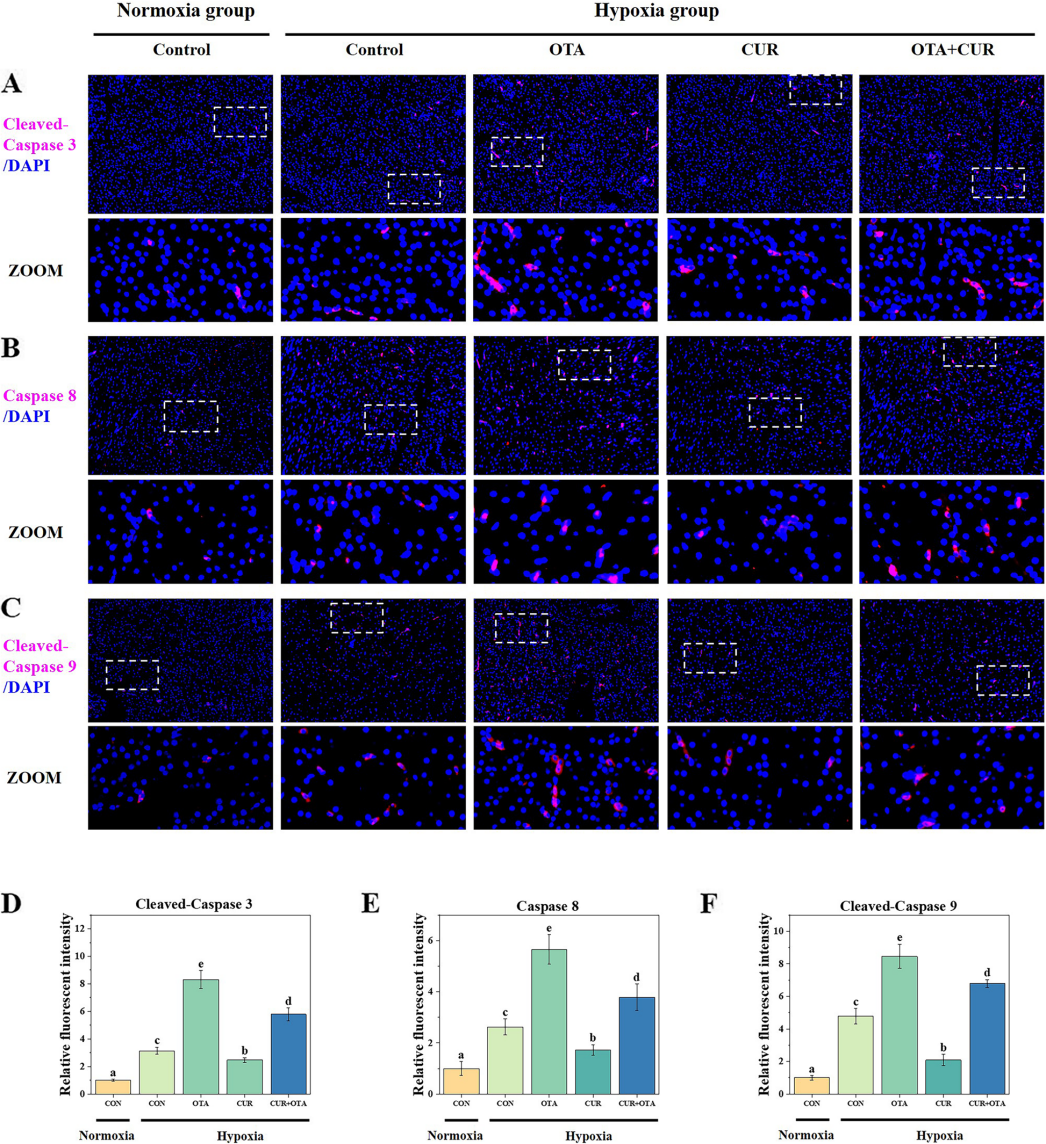
Immunofluorescence staining and quantitative analysis of liver apoptosis-related proteins (Caspase family) in grass carp. Data represent means of each group; error bars indicate SD. *n* = 6. Different letters denote significant differences between normoxia or hypoxia groups (*P* < 0.05), respectively, and * denotes significant differences between normoxia and hypoxia groups at the same level (*P* < 0.05)

**Fig. 7:**
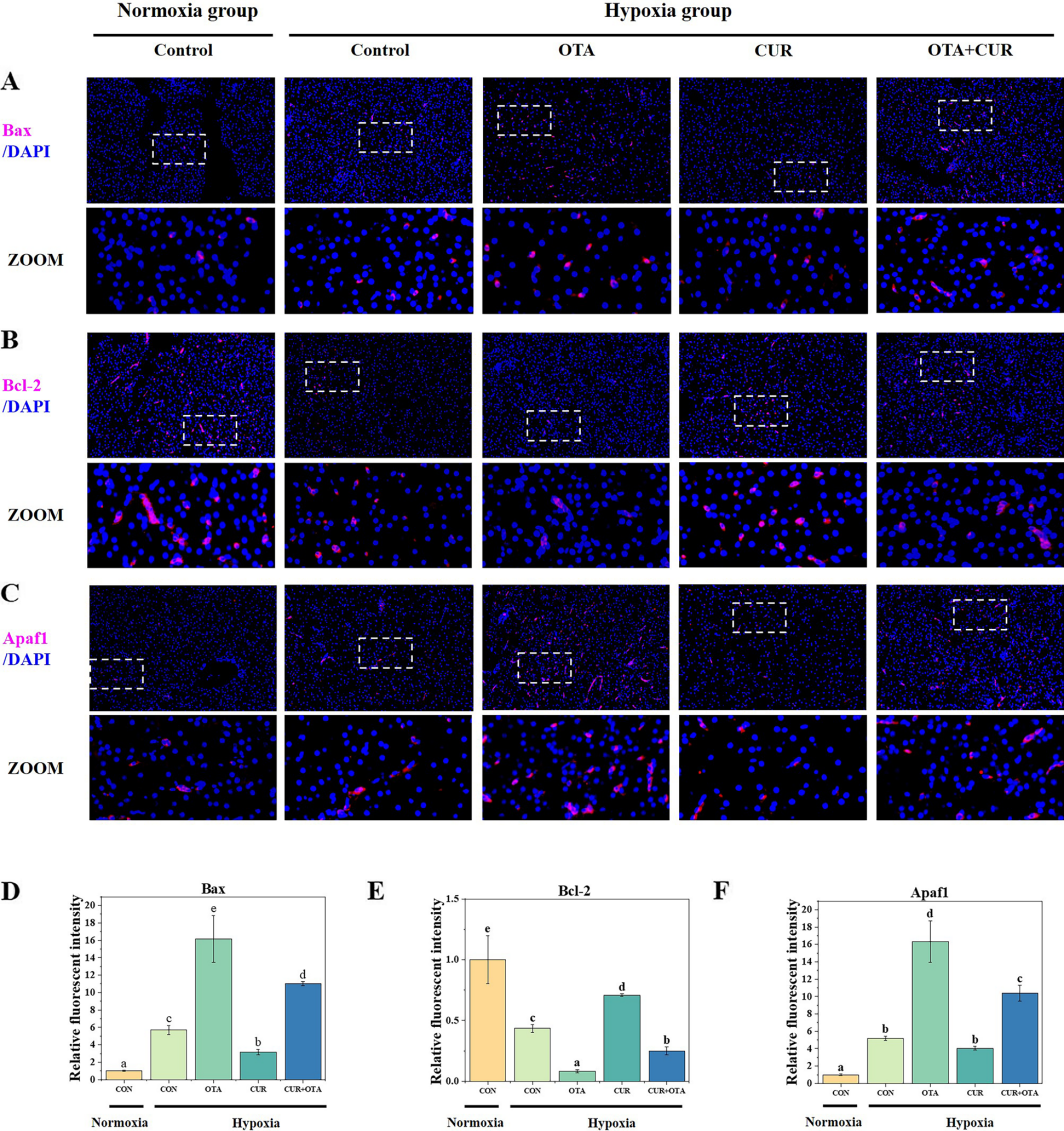
Immunofluorescence staining and quantitative analysis of liver apoptosis-related proteins (Bcl-2 family and Apaf1) in grass carp. Data represent means of each group; error bars indicate SD. *n* = 6. Different letters denote significant differences between normoxia or hypoxia groups (*P* < 0.05), respectively, and * denotes significant differences between normoxia and hypoxia groups at the same level (*P* < 0.05)

To verify that hypoxia exacerbated OTA-induced apoptosis and to investigate whether CUR could play a mitigating role, we performed a TUNEL analysis (Fig. 5A). Apoptotic cells were found to be significantly more in the hypoxia and OTA groups than that in the control group (*P* < 0.05), nevertheless, the addition of CUR significantly reduced this level (*P* < 0.05). Immediately after that, we examined the expression of apoptosis-related proteins (Fig. 5B, 6 and 7). We found that hypoxia significantly up-regulated the protein level of P53 (*P* < 0.05). Meanwhile, the co-induction of OTA and hypoxia significantly increased the expression of pro-apoptotic proteins Cleaved-Caspase 3, Caspase 8, Cleaved-Caspase 9, Bax, and Apaf1 as well as significantly decreased the expression of anti-apoptotic protein Bcl-2 (*P* < 0.05). However, the addition of CUR significantly reduced the expression of Cleaved-Caspase 3, Caspase 8, Cleaved-Caspase 9, Bax, and Apaf1, as well as significantly increased the expression of Bcl-2.

### Effect of curcumin on liver endoplasmic reticulum stress induced by the combination of ochratoxin A and hypoxia stress

**Fig. 8:**
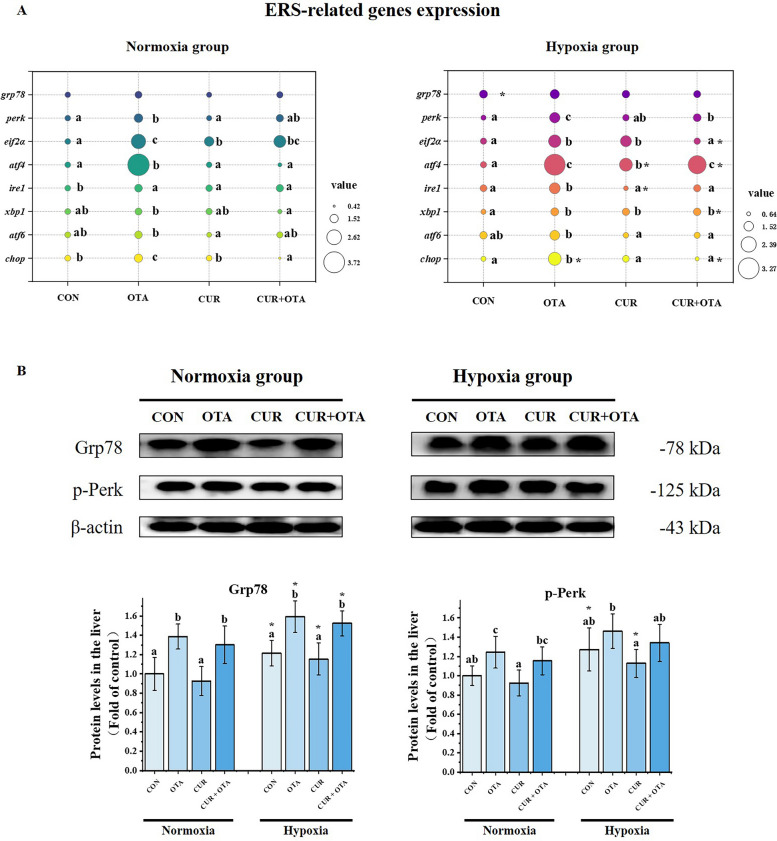
Changes in liver ERS-related mRNA (**A**) and protein (**B**) levels. The circle size represents the mean value of mRNA levels in each group. The control normoxia group was used as a reference. Data represent means of each group; error bars indicate SD. *n* = 6. Different letters denote significant differences between normoxia or hypoxia groups (*P* < 0.05), respectively, and * denotes significant differences between normoxia and hypoxia groups at the same level (*P* < 0.05)

Similarly, liver ultrastructure revealed that hypoxia exacerbated OTA-induced endoplasmic reticulum swelling. To further investigate the effect of CUR on liver ERS induced by the combination of OTA and hypoxia stress, we determined the expression of ERS-related genes and proteins. As shown in Fig. 8, hypoxia significantly up-regulated the protein expression of Grp78 (*P* < 0.05). Besides, hypoxia significantly exacerbated the OTA-induced up-regulation of mRNA levels of *chop* and protein expression of Grp78 (*P* < 0.05). However, one-way ANOVA revealed that the addition of CUR significantly down-regulated the elevation of the mRNA levels of *chop* both under normoxia and hypoxia (*P* < 0.05).

## Discussion

Ochratoxin A is widely present in various food and animal feed ingredients [28], and the liver is one of the highest target organs for OTA residue levels in grass carp [22]. Meanwhile, hypoxia is also a common environmental stressor in aquatic animals. In practice, with feed ochratoxin A contamination, aquatic animals are also often hit by the double whammy of hypoxia. However, little is known about the liver injury caused by the dual stress of OTA and hypoxia in fish. At the same time, the effect of CUR as an antioxidant on damage caused by the combination of OTA and hypoxia is not apparent. As a result, our study initially investigated the dual damaging effects of OTA and hypoxia on grass carp and the mitigating effects of CUR.

### Curcumin alleviates stress in grass carp co-induced by ochratoxin A and hypoxia

As we all know, serum Cor and Glu were considered as stress indicators to monitor the health of fish under different dietary, disease states, and environmental conditions [29]. Our study found that hypoxia exacerbated OTA-induced increases in Cor and Glu levels, suggesting exacerbated stress responses in fish. However, there are no reports on the dual effects caused by the combined effects of OTA and hypoxia on serum stress indicators in any animal. We speculate that this may be because, in toxicology, the combined action of several substances can produce more harmful effects than a single component in a mixture. Whereas the addition of CUR reduced the levels of Cor and Glu. Similarly, CUR alleviated the stress response of the gibel carp (*Carassius gibelio*) under acute ammonia stress [30]. This suggested that CUR can alleviate the stress produced in fish under the dual stress of OTA and hypoxia.

The liver is the most critical detoxification organ and the main metabolic organ in grass carp. When subjected to both OTA and hypoxia, the liver bears the brunt of the effects. Therefore, we need to investigate the effects of OTA and hypoxia on liver injury and whether CUR can reduce liver tissue damage.

### Curcumin alleviates liver tissue damage in grass carp co-induced by ochratoxin A and hypoxia

ALT and AST are important aminotransferases in animal liver [31]. In contrast, LDH is present in the cells of almost all animal organs, especially in the liver, kidney, and muscle [32]. Serum ALT, AST, and LDH are biomarkers of liver injury [33]. Our study found that hypoxia exacerbated OTA-induced increases in serum ALT, AST and LDH levels. In others’ studies, serum transaminases (ALT and AST) were significantly increased in OTA-treated rats for 14 d, indicating hepatocyte damage [34]. Besides, blood biochemical indices AST and LDH were significantly reduced in rats under hypoxia [35]. This suggests that hypoxia can exacerbate OTA-induced liver injury. Further histological analysis revealed that hypoxia exacerbated OTA-induced liver vacuolation and cellular nuclear excursions, among other things, exacerbating the damage caused to the liver. On the one hand, this may be because the liver is a target organ for OTA and hypoxia, and on the other hand, it may be because both OTA and hypoxia can cause oxidative damage in fish. Whereas the addition of CUR reduced the serum levels of ALT, AST, and LDH, suggesting that CUR alleviates OTA and hypoxia co-induced liver injury in grass carp.

Tissue damage is usually caused by oxidative damage [36]. Whereas, excessive production of ROS can cause oxidative damage [37] in animals and activate the antioxidant defense system [38]. Our study found that hypoxia exacerbated the OTA-induced increase in ROS levels and decreased the antioxidant enzymes CAT activities, and the non-enzymatic antioxidant GSH content. In contrast, the addition of CUR could reduce ROS levels and increase CAT activities and GSH content, suggesting that CUR can attenuate liver injury in fish by reducing oxidative damage and enhancing antioxidant capacity. This effect may because CUR is a free radical scavenger, so it can diffuse through the cell membrane into the cells, preventing the production of different ROS compounds [39].

### Curcumin alleviates liver mitochondrial dysfunction in grass carp co-induced by ochratoxin A and hypoxia

Mitochondria play a crucial role in cellular bioenergetic homeostasis and are closely involved in the production of adenosine triphosphate (ATP), which is intimately associated with a series of multisubunit protein complexes (complexes I–V) of the electron transport chain [40]. When the function of a particular protein complex is impaired, electrons leak from the complex, generating large amounts of ROS [41]. In the present study, hypoxia exacerbated the OTA-induced decrease in the activity of the respiratory chain complex I–V and the upregulation of ROS levels. This suggests that OTA and hypoxia disrupted the function of the mitochondrial respiratory chain complex, thereby leading to the high production of ROS. However, the addition of CUR alleviated the mitochondrial respiratory chain complex I–V expression, suggesting that CUR may ensure the activity of the complex, reduce ROS production, and ultimately alleviate liver injury. Similar results were found in other animal studies where CUR had a protective effect against OTA-induced impairment of mitochondrial function in broiler ducks [13]. This protective effect may be related to its potent antioxidant effects, as mentioned above. And Nrf2, a significant redox regulator, has a beneficial effect when mitochondrial function is impaired.

### Curcumin alleviates liver apoptosis in grass carp co-induced by ochratoxin A and hypoxia

Excess cellular levels of ROS cause damage to organelles, which can lead to apoptosis and liver damage [42]. The endogenous pathway involving mitochondria is an essential pathway in apoptosis, which leads to the release of cytochrome c, binding to Apaf1 into the formation of apoptosome and activation of the Caspase family [43]. The control and regulation of these apoptotic mitochondrial events occur through members of the Bcl-2 protein family and the tumor suppressor protein P53 has a crucial role in regulating the Bcl-2 protein family. In our study, hypoxia exacerbated OTA-induced up-regulation of pro-apoptotic-related protein expression and down-regulation of anti-apoptotic protein expression. However, the addition of CUR increased the expression of anti-apoptotic proteins and decreased the expression of pro-apoptotic proteins. Related studies have shown that CUR treatment reduced apoptosis in murine cardiomyocytes during chronic intermittent hypoxia [19]. This suggests that the addition of CUR can effectively alleviate apoptosis caused by the combination of OTA and hypoxia, which in turn alleviates the damage caused to the liver.

At the same time, the high production of ROS also leads to ERS [44], which in turn causes liver damage. We then investigated the expression of ERS-related genes and proteins.

### Curcumin alleviates liver endoplasmic reticulum stress in grass carp co-induced by ochratoxin A and hypoxia

Many external factors such as hypoxia, starvation and infection can lead to endoplasmic reticulum dysfunction and the production of large amounts of unfolded proteins, which can trigger ERS [45]. And when ERS occurs, Grp78 senses unfolded proteins, which activate three signaling pathways mediated by Ire1α, Perk and Atf6. In the first pathway, phosphorylated Ire1α could splice and activate Xbp1, which could enter the nucleus and up-regulate the expression of target genes, ultimately promoting endoplasmic reticulum-associated degradation (ERAD), protein folding, transport, and other [46]. In the second pathway, phosphorylated Perk and eukaryotic initiation factor 2 alpha (eif2α) slow down protein synthesis on the one hand and activate Atf4 and Chop on the other hand [47]. In the third pathway, activated Atf6 could promote downstream gene transcription of Grp78, EARD-related proteins, and Xbp1, among others, which together promote protein degradation [48]. In our study, hypoxia exacerbated the OTA-induced up-regulation of Grp78 expression and *chop* levels, indicating that hypoxia exacerbated OTA-induced ERS. However, the addition of CUR effectively reduced the expression of these related genes and proteins. This is consistent with experimental findings that CUR prevented chronic intermittent hypoxia-induced endoplasmic reticulum stress in murine cardiomyocytes [19]. This suggests that the addition of CUR can effectively alleviate the ERS and thus liver injury caused by the combination of OTA and hypoxia.

## Conclusion

**Fig. 9:**
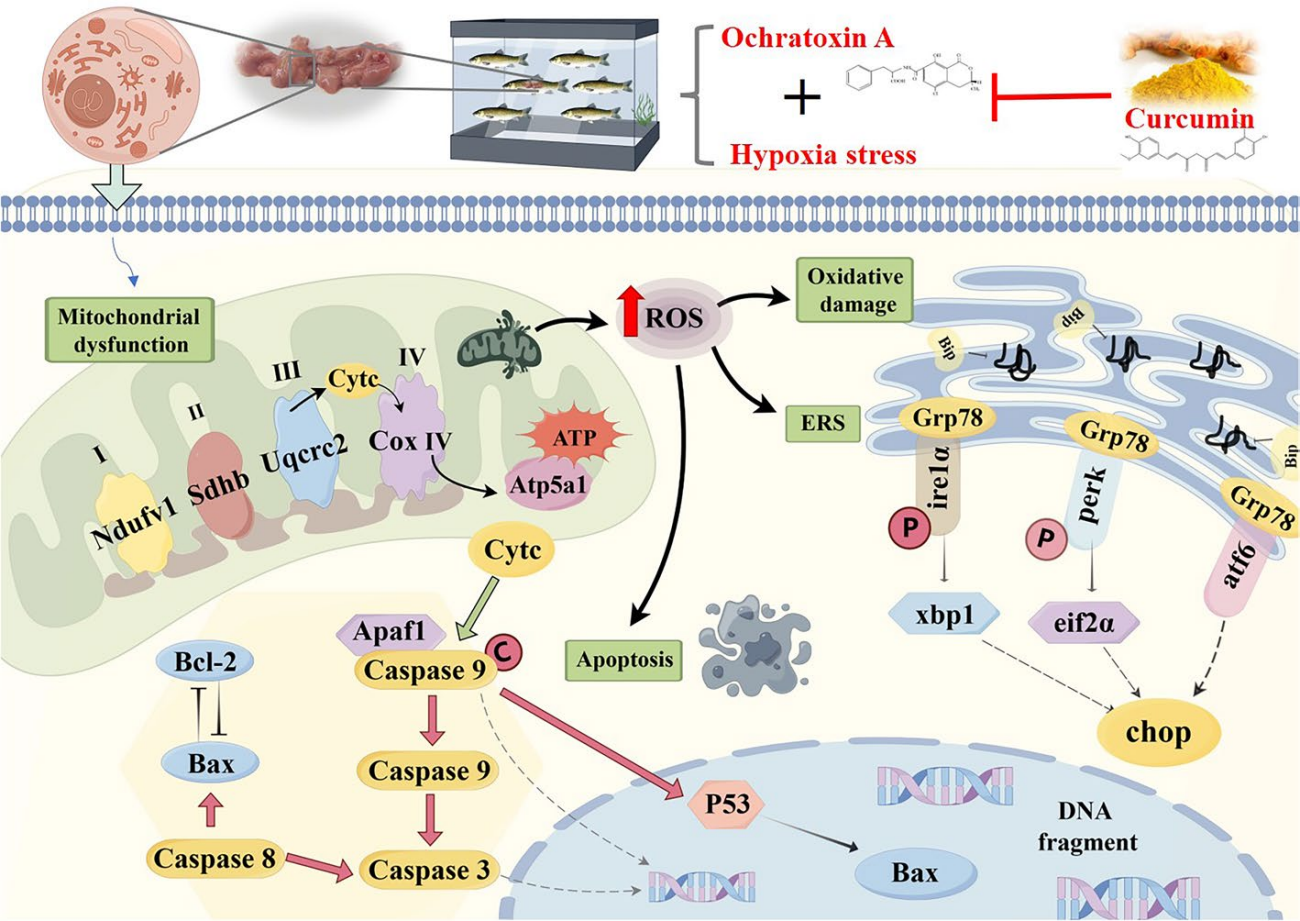
Mechanism of mitigating effects of CUR on liver biotoxicity (including mitochondrial dysfunction, oxidative damage, apoptosis, and endoplasmic reticulum stress) in grass carp caused by the combination of OTA and hypoxia. (The figure was drawn by Figdraw)

Mycotoxins are one of the greatest threats to public health, and the problem of a hypoxia environment is one of the most urgent issues that need to be solved, which are closely concerned by aquatic species. Overall, this study preliminarily investigated the mitigating effects of CUR on the biotoxicity induced by the combination of OTA and hypoxia on the liver of grass carp (*Ctenopharyngodon idellus*) (Fig. 9): (1) CUR could mitigate liver injury caused by the combination of OTA and hypoxia. (2) CUR’s ability to alleviate liver injury may be related to its inhibition of apoptosis in the mitochondrial pathway. (3) CUR’s ability to alleviate liver injury may be closely related to its ability to alleviate endoplasmic reticulum stress through three pathways: Ire1α, Perk, and Atf6. (4) CUR may reduce ROS production and oxidative stress by increasing antioxidant capacity and ensuring normal function of the mitochondrial respiratory chain complexes, which in turn reduces apoptosis and ERS. Although the current study cannot be applied to different aquatic animals, raising public awareness of harmful substances in feedstuffs and the environment is important. The results of our study will help the public to understand the mechanism of mycotoxin and hypoxia toxicity and inform the adoption of mitigation measures. Curcumin could be used as a potential natural product to mitigate the harmful effects of mycotoxins and hypoxia on production.

## Supplementary Information

**Additional file 1:** **Table S1**. The primary antibody information of Western blot analysis. **Table S2**. The primary antibody information of immunofluorescence staining. **Table S3**. The components and nutritional makeup of the basal diet. **Table S4**. The real-time PCR primer sequences.

## Data Availability

The datasets are included in this article and available from the corresponding author on reasonable request.

## Abbreviations

ALT: Alanine aminotransferase
Apaf1: Apoptotic protease activating factor1
AST: Aspartate aminotransferase
ATP: Adenosine triphosphate
Atp5a1: ATP synthase F1 subunit alpha
Bax: Bcl2 associated X protein
Bcl-2: B-cell lymphoma protein-2
Bip: Binding-immunoglobulin protein
Caspase: Cysteinyl aspartic acid-protease
CAT: Catalase
Cor: Cortisol
Cox IV: Cytochrome c oxidase
CUR: Curcumin
Cytc: Cytochrome c
DAPI: 4′,6-Diamidino-2-phenylindole
eif2α: Eukaryotic initiation factor 2α
ERAD: Endoplasmic reticulum-associated degradation
ERS: Endoplasmic reticulum stress
GLU: Glucose
Grp78: Glucose-regulated protein 78
GSH: Glutathione
Ho-1: Heme oxygenase-1
LDH: Lactate dehydrogenase
Ndufv1: NADH: Ubiquinone oxidoreductase core subunit V1
Nqo1: NAD(P)H:quinone oxidoreductase 1
OTA: Ochratoxin A
P53: Tumor protein 53
PBS: Phosphate buffered saline
Perk: Protein kinase R-like ER kinase
q-PCR: Quantitative polymerase chain reaction
ROS: Reactive oxygen species
Sdhb: Succinate dehydrogenase complex iron sulfur subunit B
SOD: Superoxide dismutase
TUNEL: Terminal deoxynucleotidyl transferase mediated dUTP nick end labeling
Uqcrc2: Ubiquinol-cytochrome c reductase core protein 2
