# Supplementary material for: Curcumin attenuates ochratoxin A and hypoxia co-induced liver injury in grass carp (Ctenopharyngodon idella) by dual targeting endoplasmic reticulum stress and apoptosis via reducing ROS content
Source: J Anim Sci Biotechnol. 2024 Oct 4;15:131. doi: 10.1186/s40104-024-01089-2 (PMC11451059; doi:10.1186/s40104-024-01089-2)
Supplement: Supplementary file 1 — Additional file 1: Table S1. The primary antibody information of Western blot analysis. Table S2. The primary antibody information of immunofluorescence staining. Table S3. The components and nutritional makeup of the basal diet. Table S4. The real-time PCR primer sequences. [file 40104_2024_1089_MOESM1_ESM.docx]

# Additional file 1

**Table S1** The primary antibody information of western blot analysis

| **Primary antibody** | **Firms** | **Product number** | **Dilution ratio** |
| --- | --- | --- | --- |
| HO-1 | ABclonal | A21452 | 1:1,000 |
| NQO1 | ABclonal | A1518 | 1:1,000 |
| GRP78 | Affinity | AF5366 | 1:1,000 |
| p-PERK | Affinity | AF4499 | 1:1,000 |
| NDUFV1 | ABclonal | A8014 | 1:500 |
| SDHB | ABclonal | A10821 | 1:500 |
| UQCRC2 | ABclonal | A4181 | 1:1,000 |
| COX Ⅳ | ABclonal | A22871 | 1:500 |
| ATP5A1 | ABclonal | A5884 | 1:1,000 |
| p53 | ABclonal | A0263 | 1:1,000 |
| Cytc | ABclonal | A13430 | 1:1,000 |

**Table S2** The primary antibody information of immunofluorescence staining

| **Primary antibody** | **Firms** | **Product number** | **Dilution ratio** |
| --- | --- | --- | --- |
| Cleaved-Caspase 3 | Affinity | AF7022 | 1:200 |
| Caspase 8 | Affinity | AF6442 | 1:200 |
| Cleaved-Caspase 9 | Affinity | AF5240 | 1:200 |
| Bax | Affinity | AF0120 | 1:200 |
| Bcl-2 | Affinity | AF6139 | 1:200 |
| Apaf1 | Affinity | AF0117 | 1:200 |

**Table S3** The components and nutritional makeup of the basal diet

| **Ingredients** | **%** | **Nutrient content** | **%** |
| --- | --- | --- | --- |
| Fish meal | 7.000 | Crude protein^5^ | 32.27 |
| Casein | 26.709 | Crude lipid^5^ | 4.98 |
| Gelatin | 6.000 | Available phosphorus | 0.84 |
| α-starch | 28.000 | n-3 polyunsaturated fatty acids | 1.04 |
| Rice flour | 15.005 | n-6 polyunsaturated fatty acids | 0.96 |
| Fish oil | 2.371 | n3:n6 | 1.08 |
| Soybean oil | 1.790 |  |  |
| Microcrystalline Cellulose | 5.000 |  |  |
| Vitamin premix^1^ | 1.000 |  |  |
| Mineral premix^2^ | 2.000 |  |  |
| Choline chloride premix^3^ | 1.000 |  |  |
| Calcium dihydrogen phosphate | 3.000 |  |  |
| BHA (99%) | 0.015 |  |  |
| L-Threonine (98.5%) | 0.110 |  |  |
| OTA and CUR premix^4^ | 1.000 |  |  |
| Total | 100.00 |  |  |

^1^Per kilogram of vitamin premix (g/kg): retiny1 acetate (1000,000 IU/g), 0.400 g; cholecalciferol (500,000 IU/g), 0.320 g; DL-a-tocopherol acetate (50%), 40.000 g; menadione (96%), 0.198 g; cyanocobalamin (1%), 0.940 g; D-biotin (2%), 0.750 g; thiamine nitrate (98%), 0.133 g; meso-inositol (97%), 22.068 g; folic acid (95%), 0.379 g; niacin (99%), 2.576 g, ascorby1 acetate (95%) and calcium-D-pantothenate (90%), 2.778 g and 4.737 g; riboflavin (80%), 0.775 g; pyridoxine hydrochloride (98%), 0.115 g. All raw materials were diluted to 1 kg with corn starch.

^2^Per kilogram of mineral premix (g/kg): Na_2_SeO_3_ (44.7% Se), 0.132 g; CuSO_4_∙5H_2_O (25.0% Cu), 0.600 g; ZnSO_4_∙H_2_O (34.5% Zn), 7.681 g; MnSO_4_∙H_2_O (31.8% Mn), 3.098 g; MgSO_4_∙H_2_O (15.0% Mg), 237.840 g; Ca (IO_3_)_2_ (3.2% I), 1.560 g; FeSO_4_∙H_2_O (30.0% Fe), 15.000 g. All raw materials were diluted to 1 kg with corn starch

^3^Choline chloride premix: choline chloride (50%), 261.95 g; the rest was diluted with corn starch to 1 kg

^4^OTA and CUR premix: Nothing was added to the control group; 1.2 mg/kg OTA was added to the OTA group; 400 mg/kg CUR was added to the CUR group; and 1.2 mg/kg OTA + 400 mg/kg CUR was added to the OTA+CUR group

^5^Crude protein and crude fat content: measured values

**Table S4** Real-time PCR primer sequences

| **Target gene** | **Primer sequence Forward (5′→3′)** | **Primer sequence Reverse (5′→3′)** | **Accession number** |
| --- | --- | --- | --- |
| *GRP78* | GTCACCTTTGAGATCGACGTG | AGAGAGTAGGCGTAGCTC | FJ436356 |
| *PERK* | CAGCGTTTACCTGGGGATGT | TTACTGCCCCAGGCGTTTAG | KX906957 |
| *ATF6* | CACCTCTGTTCCTGACCTGA | TAGACAGACAGTGGAGAGGG | KT279356 |
| *IRE1* | GAACGCCACATACTCTGA | TGTCCACTGTCACCACTA | MG797683 |
| *eIF2α* | ATCAATAGCGGAGATGGGCG | TGATGACCACCACGCATTCA | KJ126860 |
| *ATF4* | TTCGGCCAACACCTTAGACC | CTTGCCTCATCTTTCGGGGT | AY437846 |
| *XBP1s* | TTCTGAGTCCGCAGCAGGTG | GTTCTGGGTCAAGGATGTCC | KU509247 |
| *CHOP* | ATCAGAACGAGCGCCTCAAA | TTCACCTCCTGGTGTTGACG | KX013389 |
| *β-actin* | GGCTGTGCTGTCCCTGTA | GGGCATAACCCTCGTAGAT | M25013 |
